# Functional Magnetic Resonance Imaging and Obesity—Novel Ways to Seen the Unseen

**DOI:** 10.3390/jcm11123561

**Published:** 2022-06-20

**Authors:** Anna Drelich-Zbroja, Małgorzata Matuszek, Michał Kaczor, Maryla Kuczyńska

**Affiliations:** 1 Department of Interventional Radiology and Neuroradiology, Medical University of Lublin, 20-059 Lublin, Poland; zbroanna@interia.pl (A.D.-Z.); maryla.kuczynska@gmail.com (M.K.); 2 Student’s Scientific Society at the Department of Interventional Radiology and Neuroradiology, Medical University of Lublin, 20-059 Lublin, Poland; 3 Student’s Scientific Society at the Department of Endocrinology, Diabetology and Metabolic Diseases, Medical University of Lublin, 20-059 Lublin, Poland; michalkaczor@op.pl

**Keywords:** fMRI, obese, reward system, eating disorders

## Abstract

Obesity remains a pandemic of the 21st century. While there are many causes of obesity and potential treatments that are currently known, source data indicate that the number of patients is constantly increasing. Neural mechanisms have become the subject of research and there has been an introduction of functional magnetic resonance imaging in obesity-associated altered neural signaling. Functional magnetic resonance imaging has been established as the gold standard in the assessment of neuronal functions related to nutrition. Thanks to this, it has become possible to delineate those regions of the brain that show altered activity in obese individuals. An integrative review of the literature was conducted using the keywords ““functional neuroimaging” OR “functional magnetic resonance “OR “fmri” and “obesity” and “reward circuit and obesity” in PubMed and Google Scholar databases from 2017 through May 2022. Results in English and using functional magnetic resonance imaging to evaluate brain response to diet and food images were identified. The results from functional magnetic resonance imaging may help to identify relationships between neuronal mechanisms and causes of obesity. Furthermore, they may provide a substrate for etiology-based treatment and provide new opportunities for the development of obesity pharmacotherapy.

## 1. Introduction

Obesity and its numerous complications are a significant global public health problem. While there are many causes of obesity and potential treatments that are currently known, source data indicate that the number of patients is constantly increasing, which makes it a non-infectious pandemic of the 21st century. It is estimated that obesity affects nearly 40% of the world’s population and the highest prevalence is found in the United States (37.3%), Saudi Arabia (37.7%), and the United Arab Emirates (35.5%). Furthermore, regardless of social status or age, obesity is more common in women than in men [1,2]. It is alarming that obesity is also largely affecting children. According to the World Health Organization (WHO), in 2020, as many as 39 million children under the age of five had excess body weight. The actuality of the problem was also emphasized by the recent results of a study conducted in France, indicating that more than 75% of patients admitted to the intensive care unit suffering from COVID-19 infection were overweight or obese. Moreover, the risk of severe infection was three times higher in these patients compared to the population with a normal body mass index (BMI) [3]. Due to the prevalence and scale of the problem, numerous studies are constantly being conducted to understand further etiopathogenetic factors in the development of obesity at the behavioral, psychological, genetic, and environmental levels.

It is commonly believed that the development of obesity is mainly due to a disturbed energy balance when the energy intake exceeds its expenditure. Indeed, diet is seen as one of the factors that has contributed to the three-fold increase in the number of obese people in the last 40 years [1]. Undoubtedly, the type of food one chooses is important in terms of weight gain. Foods that are highly processed, high-fat, and high-sucrose (HFHS) are widely considered to be highly palatable and are therefore more likely to be eaten [4]. Such an eating pattern has been described as hedonic because the desire to eat results only from the need to feel pleasure with no energy deficit (non-homeostatic eating pattern) [5,6]. Such a view shows the involvement of the reward system in the food intake process. In addition, a chronic HFHS diet has been shown to consistently increase body weight and hyperphagia by inducing a vicious circle and promoting obesity [7]. Given these facts, it is likely that overconsumption of food eludes the individual’s consciousness.

The mechanisms above have become the subject of research and an introduction to the diagnosis of eating disorders in obese individuals. Functional magnetic resonance imaging (fMRI) has established itself as the gold standard in the assessment of neuronal functions related to nutrition. Thanks to the use of fMRI, it has become possible to delineate those regions of the brain that show different activation, e.g., in response to visual food cues. Mapping brain signaling in healthy individuals without obesity may provide a basis for defining the processes that occur in the brains of obese individuals whose food intake and nutrition mechanisms appear to be impaired. Given the fact that obesity is associated with the occurrence of numerous dangerous complications, reduced quality of life, and even increased mortality, understanding its neurophysiological mechanisms is of major importance for the effective treatment of this pathology and possible prevention of its further development. Therefore, research in this area seems to be particularly important to conduct to improve the care of obese patients.

This review aimed to examine the available fMRI evidence in the field of body–brain inter-relationships, with a particular focus on obesity and brain signaling.

## 2. Methodology

An electronic search was performed using the PubMed and Google Scholar databases. The following search terms were used: “functional neuroimaging” OR “functional magnetic resonance “OR “fmri” and “obesity” and “reward circuit and obesity”, and 1029 records were obtained. Studies published after February 2017 were included, including available full-text manuscripts in English. Additionally, the results list in the database search were manually screened for relevant and significant studies that matched the topic of the review (39 articles).

After duplicates were removed, the titles and abstracts of all records were reviewed. The decision for inclusion or exclusion of publications was made on the basis of a review of the full texts and was conducted by all authors.

## 3. Hunger and Satiety—Physiological Mechanisms

### 3.1. Food Intake Process

Food intake is under the strict control of the central nervous system and its regulation is a complex process that involves interactions between appetite and satiety factors and the brain. Both neural and endocrine factors are involved, and the process itself relies on the interaction of multiple neural pathways communicating with each other. The centers located in the hypothalamus are of the greatest importance and superiority: the hunger-related region in the lateral nuclei and the satiety-related region in the paraventricular nuclei. Antagonism in action ensures the maintenance of the body’s metabolic homeostasis and control of appetite and eating depending on energy requirements.

The hunger-related region is under the activating influence of orexigenic peptides, mainly ghrelin [8]. The increase in their concentrations in response to fasting or hypoglycemia causes direct activation of the orexigenic-sensitive regions of the hypothalamus and the release of neuropeptide Y (NPY) and agouti-related peptide (AgRP) in the arcuate nucleus, which increases appetite that promotes food hunting and intake. The opposite effect arises as a result of stimulation of the satiety-related region through the influence of anorexigenic peptides such as cholecystokinin, YY peptide (PYY), glucagon-like peptide-1, leptin, and insulin [9,10,11].

It is believed that NPY and AgRP are necessary to initiate food intake when it does not have hedonistic values (homeostatic mechanism) and hunger results only from the body’s need for energy. A study by Denis et al. conducted in an animal model showed that inhibition of the activity of NPY- and AgRP-producing neurons leads to decreased consumption of “standard” food and promotes the intake of high hedonistic and HFHS food, contributing to weight gain and obesity development [12].

The peripheral mechanism of food intake regulation is conditioned by the hormonal and nervous signaling pathways (via afferent fibers of the vagus nerve) from the gastrointestinal tract. Mainly, leptin and insulin released by adipocytes and pancreatic beta cells, respectively, are responsible for prolonged feelings of satiety. Experimental studies have shown that leptin deficiency increases appetite and leads to weight gain. It has also been proven that the amount of leptin released has a positive correlation with BMI and amount of body fat. However, obesity is characterized by impaired leptin signaling despite elevated leptin levels, or even leptin resistance resulting in no therapeutic effects of leptin administration in people with obesity.

The mechanisms above can be disrupted in obese individuals or underlie this pathology, and at the same time, they are potential points in nutritional signaling-based anti-obesity therapy that may involve ‘inhibiting’ components of the appetite pathway or ‘stimulating’ components of the satiety pathway.

### 3.2. The Role of the Reward System in Food Consumption

In the course of evolution, food itself has become considered to activate the reward circuit that is enhanced by the taste and smell of food. Nowadays, with the ubiquitous presence of highly processed foods, the consumption of HFHS food seems to be an expression of a drive for pleasure rather than an energy requirement. Hedonic feeding is governed by the reward system which is a functional unit of the limbic cortex. It is composed of closely related dopaminergic neurons of the ventral tegmental area and nucleus accumbens located in the ventral striatum. The mesocortical pathway connects the VTA with the orbitofrontal cortex, which is involved in making decisions, conscious assessment of the “pleasantness” of the stimulus, and the integration of emotional and motivational drives [6,13]. Moreover, the hippocampus plays an important role in the integration of homeostatic needs, pleasure, and motivation to act. Another significant structure is the amygdala, which is responsible for processing emotions and integrating food-related sensory signals. Although the hypothalamic hunger- and satiety-related regions, as well as the reward system and its associated structures, are presented as separate systems, they are actually closely related and cooperate in regulating food intake.

### 3.3. Other Areas of the Brain Involved in Food Processing

Functional neuroimaging studies in lean and obese individuals showed several cortical regions such as the anterior cingulate gyrus, medial prefrontal cortex, insula, posterior cingulate gyrus, temporal cortex, and orbitofrontal cortex to be differentially activated (depending on satiety level and BMI) which suggests their involvement in the regulation of food intake [14].

Insula, especially its anterior part, is known as the primary taste cortex which gathers representations of the taste, temperature, and texture of food in the mouth independently of satiety status and also of reward value and pleasantness. “Taste stimulation” in the insula is transmitted to the amygdala, where the association of feelings and emotions occurs [15].

The orbitofrontal cortex is an area of the prefrontal cortex of the brain that is involved not only in decision making and behavior inhibition but also in food stimuli processing (secondary taste cortex). It includes the conscious assessment of the “pleasantness” of the stimulus and the integration of emotional and motivational drives.

The anterior cingulate gyrus is mainly responsible for emotion processing and regulation of endocrine and autonomic responses to emotion. It is also responsible for cognitive processing, particularly reward-based decision making [15].

The amygdala exhibits extensive connections to other parts of the brain, e.g., the hippocampus and orbitofrontal cortex, which enable habit maintenance, cognitive control, and reward processing. Particularly, the connection between the amygdala and prefrontal cortex seems to be involved in the processing of subjective value, emotion regulation, and fear extinction. In some studies, the amygdala has showed greater activation in response to high-fat taste [14,15,16].

Individual differences in “reward perception” may either contribute to or result from the development of obesity. Previous studies utilizing fMRI in obesity have shown enhanced responses to visual, gustatory, and olfactory food-related stimuli in some brain regions, as well as decreased activity in others. It is believed that higher BMI might be associated with greater hypothalamic connectivity within the regions involved in food motivation and reduced connectivity with the structures associated with cognitive control of food intake. The detailed differences have been described further in the manuscripts [17,18].

**Figure 1:**
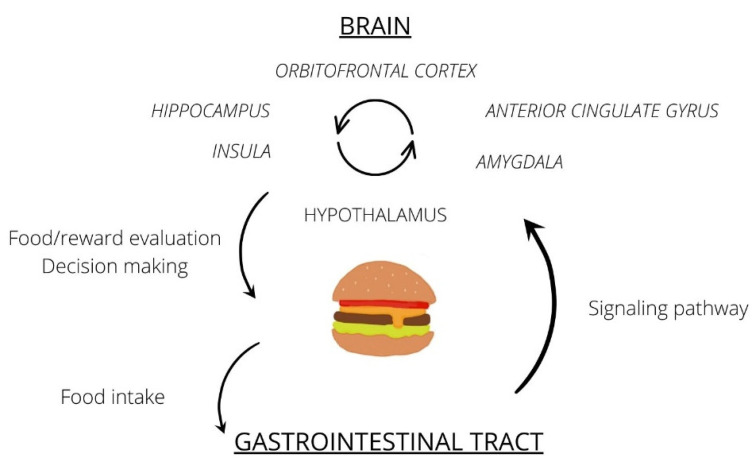
Circulation of food stimuli and engaged brain regions—simplified data.

This information is summarized in Figure 1.

## 4. Discussion

### 4.1. Food Intake in the Light of fMRI

Conventional magnetic resonance examination which most often provides information on structural conditions and brain anatomy is based on the hydrogen action in a magnetic field, while fMRI uses the magnetic properties of hemoglobin (oxidized or unoxidized) as the signal source. The fMRI examination assumes that (external—paradigm-based fMRI or internal—resting state fMRI) stimuli cause the activation of neuronal activity and connectivity, which is conditioned by an increased supply of oxygenated blood and its intensive consumption. This method is based on the differences in the magnetic properties of oxyhemoglobin and deoxyhemoglobin called blood oxygen level-dependent (BOLD) imaging, showing results in the form of brain activity maps with presentation of statistical data. Mainly, its low invasiveness has made it a valuable diagnostic tool in the examination of brain function [6]. An indisputable advantage of fMRI is the possibility of detecting pathologies that do not meet the eye of a non-infallible investigator, which do not cause alterations in the morphological image but are ongoing at the functional and biochemical level. Moreover, it is relevant that there is no need for gadolinium contrast administration, since blood acts as an endogenous, safe contrast agent [19].

Several fMRI studies have used gut hormones to delineate and distinguish areas of the brain that regulate eating behavior. Such studies have revealed a direct link between circulating intestinal hormones and neuronal activation within the regions of the brain that control appetite. Peripheral administration of the orexigenic hormone (ghrelin) has been shown to increase neuronal activation in response to visual food cues in the amygdala, prefrontal cortex, anterior cerebral hemisphere, and striatum. In turn, hypoglycemia induced by administering insulin intravenously leads to activation within the hypothalamus, cerebral hemisphere, striatum, and caudate nucleus, thereby contributing to increased appetite.

A large meta-analysis by Althubeati et al. summarized functional imaging data and collected those brain regions most extensively described in the available literature that were activated in healthy subjects in response to stimulation, e.g., glucose infusion or ingestion, PYY infusion, and standard meal intake. Areas within the cerebral hemisphere, amygdala, hippocampus, and orbitofrontal cortex were strongly associated with stimulation by hunger factors, while areas within the anterior cingulate gyrus (ACG) and striatum (putamen) showed an inverse relationship with satiety stimuli. In contrast, those brain areas that were not strongly correlated with satiety-stimulating factors were the insula, caudate cortex, thalamus, hypothalamus, OFC, and putamen [20].

### 4.2. The Influence of Obesity on the Brain

Numerous studies have reported over 200 diseases generated by obesity. Most common of them are cardiovascular diseases, type 2 diabetes, and an increased risk of neoplasms [21]. The latest reports also show the negative impact of excess body weight on the structure of brain tissue. A growing body of evidence suggests that obesity leads to changes in both the macrostructure and neuronal connections in the brain. Attention has been drawn to a link between the prevalence of obesity and the presence of cognitive and dementia disorders, along with an increased risk of developing Alzheimer’s disease [21,22]. Using morphometric methods, Raji et al. showed that in obese subjects, structural changes in the brain include the decreased volume of the frontal lobes, ACG, and hippocampus. There was also general atrophy of gray and white matter [23]. Opel et al. conducted a meta-analysis investigating the relationship between obesity (BMI > 30 kg/m^2^) and brain structure in 6420 participants. The results showed that obesity was significantly associated with cortical and subcortical abnormalities, especially in terms of lower temporo-frontal cortical thickness [24]. A reduction in the volume of subcortical structures was also observed. In terms of food processing, according to Kim et al., higher BMI was associated with greater hypothalamic connectivity to regions involved in food motivation and reduced connectivity to structures related to the cognitive control of food intake [25]. Moreover, Maayan et al. found that obese individuals have increased disinhibition and decreased cognitive control and that both of these are correlated with decreased grey matter volume in the orbitofrontal cortex [14].

Excess adipose tissue is responsible for the development of systemic inflammation affecting the whole organism. Such a mechanism is triggered by the secretory activity of overgrowth adipocytes and increased levels of circulating free fatty acids and endotoxins but also immune cells and pro-inflammatory mediators which impair the homeostasis of many organs. Such substances may cross the blood–brain barrier and induce local inflammation of the nervous tissue. Obesity-induced neuroinflammation may lead to neuronal and synaptic loss resulting in progressive cognitive decline and also to the disruption of hypothalamic satiety signals and promotion of overeating [18,26]. In addition, using magnetic resonance, obese individuals were shown to exhibit increased hypothalamic gliosis in response to ongoing inflammation that may contribute to hypothalamic leptin resistance [27]. Recent evidence supports the presence of neuroinflammation in the amygdala, hippocampus, and cerebellum [28]. Interestingly, Veronese et al. conducted a study on the effect of voluntary weight loss on the cognitive function in obese and overweight individuals. The study was conducted on 1019 participants for whom weight loss was achieved through diet, exercise, and bariatric surgery. Weight loss was found to be associated with improved attention and memory [29].

In recent years, studies utilizing fMRI have gained popularity as a way to understand the mechanisms of the development of eating disorders and obesity on the neural level, which represents a breakthrough in how obese people are considered to have altered brain signaling [18,30]. Brain activity in cortical neuronal networks involved in homeostatic control and hedonic responses is generally altered in obese individuals. In particular, decreased basal metabolism in the prefrontal cortex and striatum as well as dopaminergic alterations have been described in obese subjects with simultaneously increased activation of brain reward areas in response to palatable food cues. fMRI studies have also shown that obesity is associated with impaired brain activity within the striatum, insula, and prefrontal cortex [17].

In the study of Puzziferri et al., the authors concluded that compared to a group of lean people, women with obesity showed persistent brain activation to visual food cues even after a food intake while the lean group showed significantly decreased activation in the same areas. This included regions important for reward processing, such as the limbic cortex and midbrain [31,32]. For instance, the results of the study by Wijngaarden et al. demonstrated that food reward and food deprivation are perceived and/or processed differently by obese and lean individuals. The authors compared reward-related functional connectivity networks (hypothalamus–amygdala–cingulate cortex) via fMRI after an overnight and after a 48 h fast. The strong functional connectivity between the hypothalamus and the left hemisphere, observed at baseline, was significantly reduced in obese subjects after an extended fasting period. In contrast, after fasting, connections between the hypothalamus and the dorsal anterior cingulate cortex increased in lean subjects and decreased in obese subjects. The connection between the hypothalamus and the anterior cingulate cortex was stronger in lean subjects initially, which did not change after prolonged fasting [33].

However, it remains relatively unclear whether alterations in brain function are a cause or consequence of obesity. Therefore, Opstal et al. studied the effect of weight loss following a high-protein low-carbohydrate diet and regular physical activity on brain function in obese individuals utilizing fMRI. fMRI was performed after an overnight fast, after a prolonged 48 h fast, and after an 8 week weight loss intervention. The authors report that weight loss led to a decreased BOLD signal in the OFC and the insula, involved in feeding behavior and reward processing. These results indicate that obesity-related alterations in neuronal activity may be associated with excess body weight and may respond to body mass reduction [30].

### 4.3. Altered Reward Circuit in Obesity

“Non-homeostatic” eating is closely related to the activation of dopaminergic regions, which respond to both eating and viewing food cues [8,34]. Such an understanding of the problem implies that overeating, which leads to obesity, may be linked to pathologies within the reward system. A hyper-reactivity of the neuronal reward system, including brain areas such as the nucleus accumbens, striatum, amygdala, and orbitofrontal cortex may be the cause of the increased motivation to eat. This theory may be justified in a situation where the consumption of HFHS food is so excessive that it leads to a lower availability of dopamine receptors in obese people, in turn contributing to a reduced activation of the reward system. Such “hyper-responsivity” to pleasure may lead to seeking and consuming more and more high-calorie foods to feel pleasure and eventually results in body mass gain [8]. The “food addiction” theory states that exposure to HFHS foods modifies the brain’s reward system, leading to an addiction-like behavior pattern of compulsive overeating [35]. In the light of fMRI, it was shown that in particular, the dorsal striatum, a region commonly associated with reward anticipation and habit formation, shows increased activation in response to visual food cues in obese individuals [36]. It is still unclear whether these changes are primary or a consequence of long-term obesity, and therefore, more studies comparing brain activity maps of healthy and obese individuals are needed.

An interesting issue is the possibility of using fMRI to overlook the development of obesity in individuals with primary increased sensitivity of the reward system (predisposed ones). A study by Demos et al. that examined 58 healthy female college students over a six-month interval found that activity in the left nucleus accumbens correlated positively with changes in BMI—that greater activity was associated with greater subsequent weight gain [37]. However, such studies require a longer follow-up period, with elimination of other factors that may influence weight gain, to verify whether such a trend persists.

On the other hand, fMRI can be used to assess individual susceptibility to a particular therapy. This was proposed by Murdaugh et al. who showed that individuals with greater pre-treatment activation to high-calorie food vs. control pictures in brain reward regions, such as in the nucleus accumbens, anterior cingulate, and insula, were least successful in losing weight during the nine-month treatment [38,39]. It should be considered whether such a correlation is possible to assess, taking into account individual factors influencing weight loss (physical activity, dietary choices, and regularity of medication used, as well as genetic factors), and then tailored therapy may be implemented.

## 5. Conclusions

Obesity is characterized by distorted neural signaling and connectivity networks related to reward and perception of food-related stimuli. Undoubtedly, much remains to be discovered, but the findings of the fMRI studies give hope for the development of new tools that might be used for diagnosis, patient stratification, and etiology-based treatment personalization. Such tailored therapeutic strategies based on neural mechanisms provide hope for a lasting cure for obesity. Furthermore, they may provide a foundation for behavioral therapy for patients at the pre-obesity stage. Moreover, it is also promising in the context of childhood obesity because of fMRI safety. It appears that fMRI can also be effective in the subjective assessment of treatment efficacy.

## Data Availability

Not applicable.
